# A Patent-Pending Ointment Containing Extracts of Five Different Plants Showed Antinociceptive and Anti-Inflammatory Mechanisms in Preclinical Studies

**DOI:** 10.3390/pharmaceutics16091215

**Published:** 2024-09-17

**Authors:** Juan Carlos Barragan-Galvez, Maria Leonor Gonzalez-Rivera, Juan C. Jiménez-Cruz, Araceli Hernandez-Flores, Guadalupe de la Rosa, Martha L. Lopez-Moreno, Eunice Yañez-Barrientos, Michelle Romero-Hernández, Martha Alicia Deveze-Alvarez, Pedro Navarro-Santos, Claudia Acosta-Mata, Mario Alberto Isiordia-Espinoza, Angel Josabad Alonso-Castro

**Affiliations:** 1 Departamento de Farmacia, División de Ciencias Naturales y Exactas, Universidad de Guanajuato, Guanajuato 36200, Mexico; jcbarragang@gmail.com (J.C.B.-G.); leonor.glez.rivera@outlook.com (M.L.G.-R.); ahernandez@unsis.edu.mx (A.H.-F.); devezem@ugto.mx (M.A.D.-A.); 2 Consejo Nacional de Humanidades, Ciencias y Tecnologías (CONAHCyT)-Instituto de Investigaciones Químico Biológicas, Universidad Michoacana de San Nicolas de Hidalgo, Morelia 58030, Mexico; juan.carlos.jimenez@umich.mx (J.C.J.-C.); pnavarrosa@conahcyt.mx (P.N.-S.); 3 Instituto de Investigación Sobre la Salud Pública, Universidad de la Sierra Sur, Miahuatlán de Porfirio Díaz, Oaxaca 70800, Mexico; 4 División de Ciencias e Ingenierías, Universidad de Guanajuato, Campus León, León 37150, Mexico; delarosa@ugto.mx; 5 Chemistry Department, University of Puerto Rico at Mayagüez, 259 Boulevard Alfonso Valdez, Mayagüez 00681, Puerto Rico; martha.lopez@upr.edu; 6 Departamento de Química, División de Ciencias Naturales y Exactas, Universidad de Guanajuato, Guanajuato 36200, Mexico; eybarrientos@ugto.mx (E.Y.-B.); m.romerohernandez@ugto.mx (M.R.-H.); 7 Laboratorio de Patología Clínica, Instituto de Seguridad y Servicios Sociales de los Trabajadores del Estado (ISSSTE), Guanajuato 36000, Mexico; claucosma@hotmail.com; 8 Clinics Department, University of Guadalajara, Tepatitlan 47620, Mexico; mario.isiordia162@yahoo.com

**Keywords:** ointment, analgesics, anti-inflammatory, plant extracts, inflammatory mediators, NF-κB pathway

## Abstract

**Background/Objectives**: The antinociceptive and anti-inflammatory effects of a patent-pending ointment containing plant extracts from *Eucalyptus globulus*, *Curcuma longa*, *Hamamelis virginiana*, *Echinacea purpurea*, and *Zingiber officinale* were evaluated. **Methods**: Plant extracts were chemically characterized by gas chromatography–mass spectroscopy. The antinociceptive activity of the ointment was assessed using the hot plate, tail flick, and formalin tests, whereas the anti-inflammatory activity was measured using the acute and chronic TPA-induced ear edema tests. Mechanisms of action were evaluated using inhibitors from signaling pathways related to pain response and by using histological analysis and assessing the expression and activity of pro-inflammatory mediators. **Results**: The ointment showed antinociceptive and anti-inflammatory effects like those observed with diclofenac gel (1.16% *v*/*v*) and ketoprofen gel (2.5% *v*/*v*). The antinociceptive actions of the ointment are mediated by the possible participation of the opiodergic system and the nitric oxide pathway. The anti-inflammatory response was characterized by a decrease in myeloperoxidase (MPO) activity and by a reduction in ear swelling and monocyte infiltration in the acute inflammation model. In the chronic model, the mechanism of action relied on a decrease in pro-inflammatory mediators such as COX-2, IL-1β, TNF-α, and MPO. An in-silico study with myristic acid, one of the compounds identified in the ointment’s plant mixture, corroborated the in vivo results. **Conclusions**: The ointment showed antinociceptive activities mediated by the decrease in COX-2 and NO levels, and anti-inflammatory activity due to the reduction in IL-1β and TNFα levels, a reduction in MPO activity, and a decrease in NF-κB and COX-2 expression.

## 1. Introduction

Different synthetic steroidal and non-steroidal drugs are used to treat inflammation and pain in patients. In some cases, these drugs produce a variety of side effects, including gastric ulcers, kidney failure, and liver damage, among others [1]. Compounds derived from medicinal plants represent an alternative to treat pain and inflammation-causing conditions, such as the alkaloid morphine, isolated from *Papaver somniferum* L. (Papaveraceae); capsaicin, obtained from some *Capsicum* species (*Capsicum annuum* L. (Solanaceae) and *Capsicum chinense* Jacq (Solanaceae)); and salicylic acid, a molecule extracted from *Salix alba* L. (Salicaceae) [2].

Preparation of gels, ointments, and creams with plant extracts has been reported. Different research groups have also studied the effects of these preparations. For instance, *Eucalyptus globulus* Labill (Myrtaceae) (EG) oil (0.2% *v*/*v*) suppressed inflammation in LPS-induced murine lung alveolar macrophages through the NLRP3 inflammasome and the MAPK pathways [3]. *Eucalyptus globulus* essential oil (11.5–45 mg/kg i.p.) decreased antinociception in a similar pattern to morphine (4 mg/kg i.p.) by the possible participation of the µ-opioid pathway in C57BL/6 mice [4]. In clinical trials, it was proven that *Curcuma longa* L. (Zingiberaceae) (CL) extracts acted as non-steroidal anti-inflammatory drugs (NSAIDs) and were able to reduce osteoarthritis or obesity-associated inflammation [5,6]. Also, *Hamamelis virginiana* L. (Hamamelidaceae) (HV) decreased inflammation in HaCaT cells infected with *Cutibacterium acnes* by decreasing IL-6 levels (IC_50_ = 136.90 ± 17.22 μg/mL) and NF-κB-driven transcription (IC_50_ = 136.90 ± 17.22 μg/mL) [7]. An ethanol extract of *Echinacea purpurea* (L.) Moench (Asteraceae) (EP) at 1–3 g/kg reduced inflammation in an ulcerative colitis model induced by 2,4,6-trinitrobenzene sulfonic acid in rats by decreasing the NLRP3 inflammasome pathway and the levels of IL-6 and TNF-α [8]. An ethanol extract of ginger (*Zingiber officinale* Roscoe (Zingiberaceae) [ZO]) rhizome (100–400 mg/kg p.o.) decreased cardiac fibrosis by lowering the expression level of transforming growth factor (TGF)-β1 and TGF-β3 in streptozotocin-induced diabetic rats [9].

Plant-derived molecules are already used in pharmaceutical products available on the market. For example, capsaicin, isolated from *Capsicum annum*, is contained in the creams Capzasin-P^®^ (Chattem Labs, Chattanooga, TN, USA) and Zostrix-HP^®^ (Rafa Laboratories, Jerusalem, Israel). Herein, the formulation contains 0.025% capsaicin (weight/weight). Flavocoxid (Limbrel^®^), used for treating rheumatoid arthritis, contains the flavonoids baicalin and catechin obtained from *Scutellaria baicalensis* and *Acacia catechu*.

Topical formulations such as ointment do not cause gastric, kidney, or liver damage. Therefore, they are an attractive option for the treatment of localized pain. The objectives of the present work were (a) to perform a chemical standardization using GC-MS of the plant mixture containing EG, CL, HV, EP, and ZO extracts; (b) to evaluate the antinociceptive and anti-inflammatory activities of a proprietary ointment; (c) to assess the molecular mechanisms of action of the anti-inflammatory and antinociceptive effects of the ointment; and (d) to perform an in-silico study of the interaction of some compounds found in the plant extract mixture with antinociceptive and anti-inflammatory protein targets. 

## 2. Materials and Methods

### 2.1. Gas Chromatography–Mass Spectrometry Analysis

The mass chromatography procedure for the chemical standardization of plant extracts was described previously [10]. 

### 2.2. Animals

Male BALB/c mice, weighing 25 ± 5 g and 4–6 weeks old, and male Wistar rats, weighing 250 ± 20 g and 8–10 weeks old, were obtained from the University of Guanajuato bioterium. The rodents were housed in isolated cages at 24 °C under a light–dark cycle of 12:12 h and supplied with food and water ad libitum. Before experiments were initiated, experimental protocols were approved by the Institutional Committee on Bioethics in Research from the University of Guanajuato (CIBIUG-P10-2022). Protocols complied with the Official Mexican Norm NOM 062-ZOO-1999 (technical specifications for the production, care, and use of laboratory animals).

### 2.3. Drugs and Reagents

Diclofenac (1.16% *w*/*w*) and ketoprofen (2.5% *w*/*w*) gels under the commercial names of Fastufrem^®^ and Voltaren^®^, respectively, were purchased from a drugstore. In addition, 12-O-tetradecanoylphorbol-13-acetate (TPA), formalin, acetone, NG-nitro-L-arginine methyl ester (L-NAME), glibenclamide, naloxone, sodium chloride (NaCl), disodium hydrogen phosphate (Na_2_HPO_4_), sodium phosphate (NaHPO_4_), sodium biphosphate (NaH_2_PO_4_), hydrogen peroxide (H_2_O_2_), Bradford reagent, albumin, tetramethylbenzidine HCl, sodium acetate, and dimethylformamide were acquired from Sigma-Aldrich (St. Louis, MO, USA). The anti-nuclear factor-κB (NF-κB) p65 (#51-0500), anti- NF-κB p65(Ser536) (T.849.2), anti-Cyclooxygenase2 (COX-2) (PA1-9032), anti-beta actin (BA3R), and anti-rabbit IgG (#31460) antibodies were purchased from Thermo Fisher Scientific (Cleveland, OH, USA).

### 2.4. Plant Material

Plant samples were obtained from Starwest Botanicals (Sacramento, CA, USA) in May 2020. Dr. Eleazar Carranza from the herbarium Isidro Palacios (SLPM) identified plant samples with the following voucher numbers: *Eucalyptus globulus* Labill. (Myrtaceae) (SLPM 60722) (EG), *Curcuma longa* L. (Zingiberaceae) (SLPM 50048) (CL), *Hamamelis virginiana* L. (Hamamelidaceae) (SLP 23635) (HV), *Echinacea purpurea* (L.) Moench (Asteraceae) (SLPM 31454) (EP), and *Zingiber officinale* Roscoe (Zingiberaceae) (SLPM 50049) (ZO).

### 2.5. Plant Extract Preparation

A patent-pending method (MX/a/2024/008251) to obtain the plant extracts was used. Briefly, EG, CL, HV, EP, and ZO tissues were used for extract preparation. The plants were dried at room temperature. The plants were placed in a glass beaker and mixed with organic olive oil at the ratios shown in parentheses: (a) EG leaves (1.63% *w*/*v*), (b) CL roots (7.64% *w*/*v*), (c) HV leaves (1.55% *w*/*v*), EP roots (1.55% *w*/*v*), and ZO roots (7.64% *w*/*v*). The selection of plant rations was based on preliminary works carried out in the laboratory. Later, the mixture was heated at 60 °C for 8 h with manual stirring every 30 min. Then, the solution was cooled down to room temperature, and oil extracts were separated by filtration.

### 2.6. Ointment Preparation

As stated in MX/a/2024/008251, a homogenous mixture of 2510 mL of hemp seed oil, 564 g of organic wax previously melted in a water bath, and 920 mL of plant extract were obtained. The mixture was allowed to cool at room temperature.

### 2.7. Ointment Physicochemical and Organoleptic Characterization

Organoleptic characteristics (color, smell, and consistency) were evaluated after 24 h and 6 months of ointment preparation. Physicochemical properties (centrifugation test, pH, and extensibility test) were assessed following the Mexican regulation (NOM-073-SSA1-2005) to evaluate the ointment stability [11]. One gram of ointment was carefully weighed and dispersed in 10 mL of purified water. Ointment pH was measured using a pH meter after 48 h, 1 week, 2 weeks, 1 month, 3 months, and 6 months after preparation. For the extensibility test, 2 g ointment were placed at the center of a glass plate. Another glass plate was homogeneously dropped over the ointment plate at 0.5 cm. The sample was allowed to expand for 5 min before the diameter of the sample expansion was measured. For the centrifugation test, one gram of ointment was centrifugated at 800× *g* for 30 min at room temperature. After centrifugation, the ointment was visually examined for potential changes. 

### 2.8. Skin Irritation Test

OECD guideline number 434 protocol was followed to evaluate the acute dermal toxicity of the ointment [12]. Two experimental groups (n = 5 in each group) were used. Twenty-four hours before the experiment, 10% of the dorsal zone was shaved in each animal. Once a day for 14 days, samples of 50 mg of ointment with extracts and without extract (vehicle) were applied to the shaved area of the rats. The animals were observed for any adverse skin reaction (edema or erythema).

### 2.9. Formalin Test (FT)

A formalin-induced nociception test was performed as described previously [13]. Groups of mice (n = 8 per group) were treated topically with 10 mg of vehicle (ointment without plant extracts), 10 mg of ointment with plant extracts, or diclofenac gel (1.16% *w*/*w*) as positive control. The selection of a single concentration was based on previous studies carried out in our laboratory. Preliminary results indicate that this concentration showed the highest anti-inflammatory and antinociceptive activity. No more concentrations were reported to decrease the number of animals used in this study. Sixty minutes after the administration of treatments, the mice were injected with 30 µL of 3% formalin into the subplantar space of the right hind paw and then placed in acrylic cylinders. The time spent by the mice licking the paw was recorded to identify two phases: (a) 0–15 min indicates neurogenic pain, and (b) 15–45 min relates to inflammatory pain. Antinociception was expressed as the percentage of inhibition in licking response or biting compared to the vehicle group.

A potential mechanism of the antinociceptive effects of the ointment was determined through an intraperitoneal injection with 5 mg/kg of naloxone, 20 mg/kg of NG-nitro-L-arginine methyl ester (L-NAME), or 10 mg/kg of glibenclamide. Each inhibitor dose used in this study was based on previous work in our research group [14]. Fifteen minutes after injection administration, the ointment was applied to the subplantar space. In addition, after 45 min, mice received 3% formalin (30 µL), and the test was carried out as described previously.

### 2.10. Tail Flick Test (TFT)

The TFT is a spinal response test [15]. The equipment calibration was performed by a specialist technician seven months before performing the nociceptive tests. Non-treated mice were used as controls during the calibration of the tail flick apparatus. The animal habituation was performed following the protocol indicated by Minett et al. [15].

Experimental groups consisted of 10 mg of vehicle (ointment without plant extracts), 2.5% *w*/*w* ketoprofen gel, or ointment containing plant extracts (10 mg) topically administered to the tails of three groups of mice (n = 8 per group). TFT was applied to each mouse group according to [16]. Briefly, the distal half of each mouse tail was extended on a metal surface. Later, radiant heat emitted from the analgesia meter was provided as a noxious stimulus to the tail. Discomfort signs were defined as the withdrawal of the tail from light–heat exposure. Latency (seconds) measured the time it took the mice to withdraw the tail from the light–heat source. The evaluation was performed at 60 and 120 min after dermal administration of vehicle, ketoprofen, or ointment. Mice that exhibited latency values between 4 and 8 s were selected for the tail flick test, and a cut-off time of 16 s was set to prevent tail injury.

### 2.11. Hot Plate Test (HPT)

The HPT is a supraspinal thermal assay [15]. The equipment calibration and the animal habituation were performed as described in Section 2.10. The HPT was carried out on a hot metal plate at a temperature of 55 ± 1 °C according to Turner’s method [17]. First, mice with a latency time of 3–8 s were selected for the test. Herein, the hind paws of three groups of mice (n = 8) received topical administration of vehicle (10 mg), ketoprofen gel (2.5%), or ointment (10 mg). Latency time (seconds) was recorded when acute discomfort appeared after the mice were placed on the hot plate. Discomfort signs include licking the hind paw or jumping to escape from the heat source. A cut-off time of 30 s to avoid injury was selected. This test was performed 60 and 120 min after dermal administration.

### 2.12. TPA-Induced Acute and Chronic Mouse Ear Edema

Acute edema was induced as previously described by De Young et al. [18]. Briefly, 2.5 µg of TPA (in 20 µL of acetone) were administered to the internal and external surface of the mice’s right ear. Sixty min before TPA application, three groups (n = 8 per group) were treated with 10 mg of ointment, 10 mg of diclofenac gel (1.16% *w*/*w*), or the vehicle group (ointment without plant extracts). After 6 h, the mice were sacrificed by cervical dislocation, and 6 mm-diameter ear biopsies were obtained with a punch, weighed, and stained with hematoxylin–eosin (H&E).

The chronic ear edema test was carried out as described previously [19]. Three groups (n = 8 per group) were treated with TPA (2.5 µg in 20 µL acetone) as described above, and after 40 min, each group was treated by topical administration with 10 mg of ointment and diclofenac gel (2.3% *w*/*w*). Topical treatment and TPA application were performed every 48 h for 10 days. On the last day of the experiment, the mice were sacrificed, and biopsies were obtained as described above and stained with H&E. The percentage of anti-inflammatory effect for acute and chronic edema was determined as previously described [20].

### 2.13. Tissue Homogenization

Circular ear biopsies from the mice and hind paw of mice in the formalin test were placed in tubes with Pierce^®^ IP lysis buffer and homogenized by a BeadBug^TM^ homogenizer using zirconium beads. Each homogenate was centrifuged for 5000× *g*/15 min, and the supernatant was saved for further determinations.

### 2.14. Protein Quantification by Bradford Assay

Before protein quantification, 1 μL of the homogenate was diluted with 40 μL of phosphate buffer with a pH of 7.4 (150 mM NaCl and 10 mM Na_2_HPO_4_) to eliminate any interference generated by the lysis buffer used. Subsequently, 20 μL of diluted homogenate were mixed with 200 μL of Bradford Reagent (Pierce TM Bradford Plus Protein Assay Reagent). Samples were analyzed with a 630 filter without a differentiating filter using the ELISA reader (CHROMATE AWARENESS TECHNOLOGY INC., Palm City, FL, USA). The range of the albumin calibration curve for sample quantification was 40 to 210 μg/mL. This methodology was based on Ref. [21].

### 2.15. Enzyme-Linked Immunosorbent Assay (ELISA)

The TPA-induced chronic ear edema test homogenates were diluted 3 or 5 times with the ELISA sample diluent buffer for TNF-α or IL-1β, respectively. Then, 100 µL of diluted homogenate were added to mouse IL-1β or a TNF-α antibody-coated ELISA plate (KIT TNF-α (RAB0477A-EA) or IL-1β (RAB0275), Sigma-Aldrich) and incubated at room temperature for 2.5 h with shaking at 180 rpm (YANKEE ROTOR CLAY ADAMS, Parsippany, NJ, USA). The solution was discarded and washed 4 times with 300 µL 1× washing buffer. Then, 100 µL biotinylated mouse IL-1β or TNF-α detection antibodies were added and incubated at room temperature for 1 h with shaking at 180 rpm. The solution was discarded and washed 4 times with 1× washing buffer, and 100 µL of HRP-streptavidin were added and incubated at room temperature for 45 min. Then, the reaction was washed 4 times with 1× washing buffer (1 wash per minute), and 100 µL of ELISA colorimetric TMB reagent (HRP Substrate) was added and incubated at room temperature for 30 min while protected from light. Finally, 50 µL of ELISA stop solution were added to each well, and the plates were read immediately at 450 nm. The homogenates were quantified using calibration curves of TNF-α and IL-1β in the ranges of 93.73 to 6000 and 2.74 to 666.7 pg/mL, respectively. 

### 2.16. Myeloperoxidase (MPO) Assay

MPO assay was performed according to De Young et al. [18] and Suzuki et al. [22] with minor modifications. Thirty microliters of ear tissue homogenate (6 mm) were mixed with 100 μL of phosphate buffer pH 7.4 (150 mM NaCl and 10 mM Na_2_HPO_4_), 85 μL of 0.22 M phosphate solution pH 5.4 (NaHPO_4_ 216.6 mM and 3.42 mM NaH_2_PO_4_), and 15 μL of 0.017% H_2_O_2_ in a 96-well microplate. The reaction started with the addition of 30 μL of 18.4 mM tetramethylbenzidine HCl prepared in 8% aqueous dimethylformamide. The resulting mixture was incubated at 37 °C for 3 min using a thermal bath (WB100-1). The reaction was stopped by adding 30 μL of 1.46 M sodium acetate at pH 3.0. Finally, myeloperoxidase activity was determined using a CHROMATE-4300 ELISA reader (AWARENESS TECHNOLOGY INC., Palm City, FL, USA) with 630 primary filters without a differentiating filter. Results were reported as mOD/mg of protein.

### 2.17. Nitric Oxide (NO) Estimation

The nitric oxide (NO) levels in the homogenates from the hind paw of mice in the formalin test were determined using the Griess reagent (1% sulfanilamide in phosphoric acid). Homogenates (50 μL) were collected and mixed with Griess reagent (50 μL), followed by incubation at room temperature for 10 min while protected from light. Then, 50 μL N-(1-Naphthyl) ethylenediamine dihydrochloride, prepared in distilled water, were added. The reaction mixture was incubated at room temperature for 10 min and protected from light. The optical density was measured at 492 nm using a CHROMATE-4300 ELISA reader (AWARENESS TECHNOLOGY INC., Palm City, FL, USA). The nitrite concentration was determined by extrapolation from the sodium nitrite standard curve (7–100 µM).

### 2.18. Western Blot Analysis

Protein extractions from lysate samples were resolved by 12% sodium dodecyl sulfate–polyacrylamide gel electrophoresis (SDS-PAGE) under reducing conditions. Proteins were electro-transferred to a polyvinylidene difluoride (PVDF) membrane stained with Ponceau Red solution (cat A40000278, Thermo Fisher) and then incubated with primary antibodies diluted at 1:500 and with a secondary antibody at 1:10,000 using an iBindTM Flex Western device (Thermo-Fisher Scientific) following the manufacturer’s protocol. The membrane was revealed using SuperSignal™ West Femto ECL and visualized in the ChemiDocTM imaging system (Biorad, Hercules, CA, USA). The intensity of the bands on the blots was quantified using ImageJ software (version 1.54j) and normalized for beta-actin values as a loading control for densitometry analysis.

### 2.19. In-Silico Study, Computational Details

The in-silico study began with a conformational search for ligands (myristic acid, palmitic acid, valeric acid, and stearic acid) using Spartan’20 software [23]. These ligands were some of the compounds found in the plant mixture contained in the ointment. The conformer with the highest population contribution was selected by calculating its optimized geometry using Gaussian 16 software [24] at the B3LYP/6-311G level of theory. The molecular docking study began after the optimization of the ligands. The antagonist β-funaltrexamine (PDB ID: 4DKL) and the reference drug ethilisothiourea (ITU) (PDB ID:4NOS) were used for the analysis of the μ-opioid receptor and the nitric oxide synthase protein, respectively. Water molecules and other ligands were removed from each protein subunit, and the macromolecule was prepared using the UCSF Chimera software version 1.16 [25]. Ion charge and metal (Fe and Zn) parameters were assigned in the iNOS protein to prepare the receptors. Molecular docking was performed with 100 runs in AutoDock 4.2 [26] using the default parameters with a population of 1500 individuals. Discovery Studio software version 21.1.0 [27] allowed the visualization of the molecular docking poses. The stability and evolution of the drug–receptor interaction concerning the time of the best poses obtained in the molecular docking were analyzed through molecular dynamics simulations using the NAMD software version 3.0 [28]. The modules CHARMM-GUI [29] and Solution Builder [30] and the CHARMM36 force field [31] analyzed the complex parameterization. The pH was set to 7.4 in the generated cubic cell, and the solvation was periodically applied with the TIP3 water model. NaCl ions were added at 0.15 M to guarantee the system neutrality, whereas the particle mesh Ewald (PME) method [32] modeled the long-range electrostatic interactions. For the simulation protocol, periodic conditions avoided boundary effects, and the conjugate gradient algorithm reduced conflicting contacts. The system was then heated from 0 to 310 K for 500 ps, maintaining the temperature for 500 ps using the NVT assembly and employing the Langevin thermostat [33]. The system was kept in equilibrium for 5 ns within the NPT assembly (1 atm and 310 K). Subsequently, the production was carried out for 100 ns integrated by 2 fs time steps. The molecular dynamics simulation results were analyzed by root mean square deviation (RMSD), and VMD software version 1.9.4 [34] calculated hydrogen bond interactions. The Poisson–Boltzmann molecular mechanics surface area, using gmx_MMPBSA [35], estimated the binding free energy from the molecular dynamics simulation. The last 40 ns of the simulations at the production stage were considered for the analysis.

### 2.20. Statistical Analysis

All experimental values are expressed as the mean ± standard error. Statistically significant differences from the vehicle group were identified by Student’s *t*-test or ANOVA with the post hoc Tukey test for paired data. A level of *p* ≤ 0.05 was used to determine statistical significance. All calculations were performed using the Graph Pad Prism V.8 software system (GraphPad Software, San Diego, CA, USA).

## 3. Results

### 3.1. Chemical Composition of the Ointment

**Figure 1:**
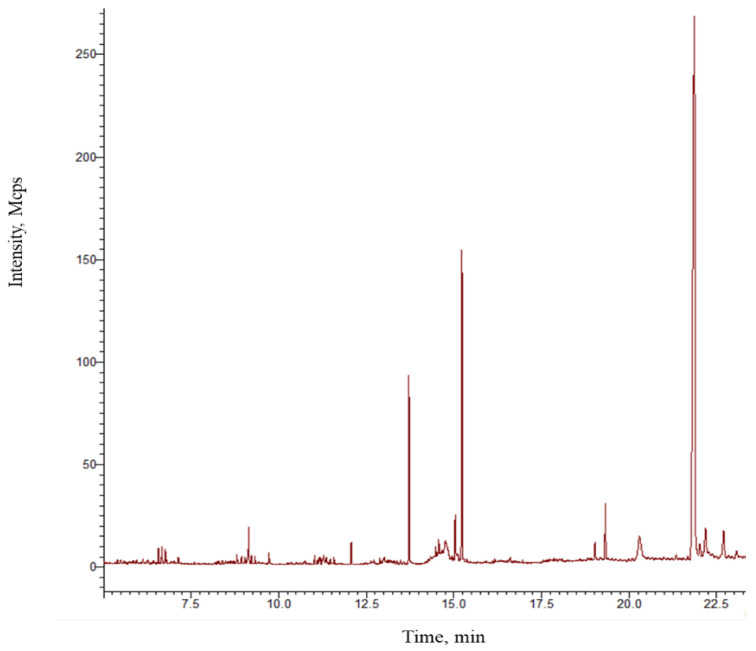
GC-MS chromatogram of the ointment prepared with plant extracts. Peaks correspond to the compounds identified and listed in Table 1.

The plant metabolites in the extracts used in the ointment preparation were identified using gas chromatography–mass spectrometry (GC-MS). A total of 26 different compounds were determined (Table 1). Results indicate that most compounds corresponded to fatty acid esters (FAEs). Three peaks with strong signal intensity were observed in the chromatogram (Figure 1), corresponding to palmitic acid, stearic acid, and an oily compound (acetoxy–cholest). Other molecules with low-intensity signals were also identified as fatty acids (FAs), and they included docosene, oleic acid, squalene, and heneicosane, as well as hydroxyfatty acids such as trimethylsilyl heptadocaenate.

### 3.2. Organoleptic and Physicochemical Characterization

The ointment aspect was greasy, yellow, and with a plant odor. Six months post preparation, the vehicle and the ointment showed no changes in color, odor, or appearance. In addition, no agglomeration or phase separation was observed. After the centrifugation test, formulations exhibited plasticity and stability. Samples showed a pH of 6.00 after preparation, and the pH decreased to 5.60 after 6 months of ointment production. 

### 3.3. Skin Irritation Test

After 14 days of topical application to the mice, the vehicle and the ointment produced no inflammation, erythema, or edema.

### 3.4. Antinociceptive Response 

**Figure 2:**
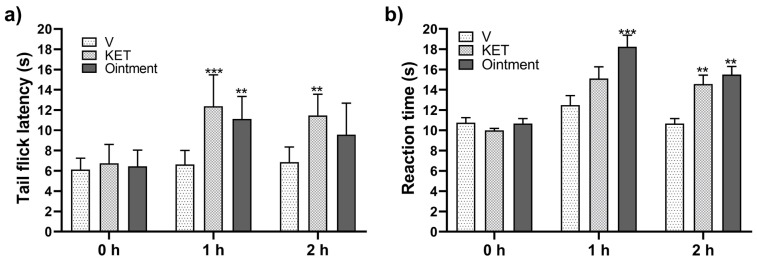
Results of thermal nociception induced by the TFT (**a**) and HPT (**b**). The antinociceptive effect was calculated as the reaction time (seconds) of the mice to heat-induced pain at 0, 1, and 2 h after topical application of the ointment. The vehicle group (V) refers to mice treated with saline solution only. Ketoprofen gel (2.5% *w*/*w*) (KET) was used as the reference drug. Bars represent mean values (±SEM) for experimental groups (n = 9), ** *p* < 0.01, *** *p* < 0.001, compared to vehicle.

The ointment showed an antinociceptive effect in TFT by increasing (*p* < 0.05) latency time compared to the vehicle (Figure 2a). Topical administration of plant-based ointment onto the mice’s paws 1 h and 2 h before the HPT significantly increased (*p* < 0.05) latency time against heat-induced nociception compared to that observed when the vehicle (ointment without plant extracts) was applied (Figure 2b). The reference drug ketoprofen (2.5% *w*/*w*) and the plant-based ointment were applied 1 h and 2 h before the HPT. The plant-based ointment produced a significantly higher latency time when pretreatment was performed 1 h before the HPT. No significant differences between ketoprofen and ointment were observed when the HPT was conducted 2 h after product application (Figure 2b). The ointment showed a similar antinociceptive effect as that shown by ketoprofen at 1 and 2 h post treatment in the TFT and HPT (Figure 2).

**Figure 3:**
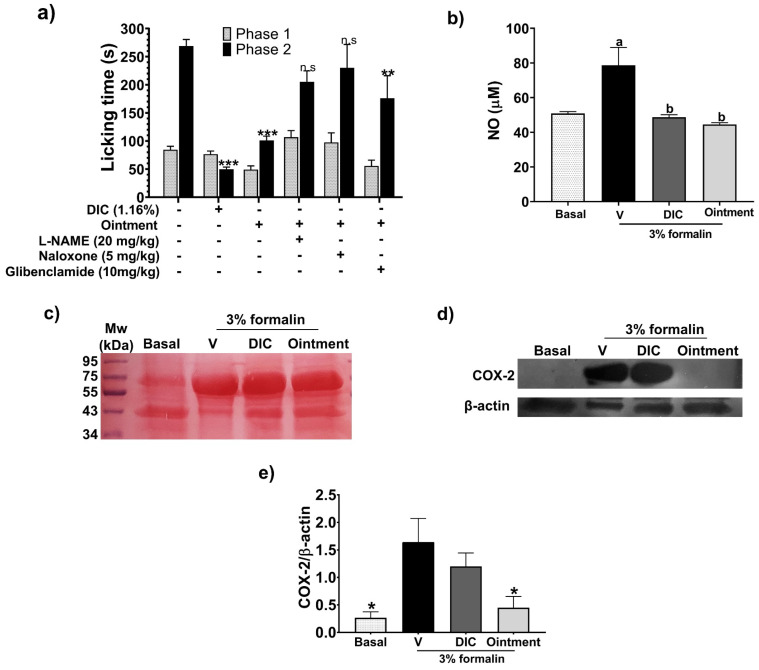
Ointment’s antinociceptive effect and potential mechanism. (**a**) The ointment’s pharmacological effect against formalin-induced pain was measured as cumulative licking time (seconds) of mice. Homogenates from the hind paws of the mice were used to measure nitric oxide (NO) production (µM) (**b**) and electrotransferred to PVDF membrane stained with Ponceau red (**c**) and immunoblotted with anti-COX2 and anti-β-actin antibodies for densitometric analysis (**d**,**e**). The ointment was applied topically 1 h before the intradermal application of formalin. Diclofenac (DIC) 1.16% gel was used as a positive control. The vehicle group (V) was treated with saline solution, and the basal group was the untreated left hind paw. Bars represent mean values (±SEM) for experimental groups (n = 9), ** *p* < 0.01, *** *p* < 0.001, compared to the vehicle group (columns 1–2) in (**a**); a and b mean *p* < 0.05 compared to the basal and vehicle group, respectively, in (**b**); and * *p* < 0.05 compared to the vehicle group in (**e**).

Plant-based ointment significantly decreased licking time in phase 2 (15–45 min) of the FT compared to that observed with the vehicle group (no ointment application). On the other hand, no significant effect was observed in phase 1 (Figure 3, columns 5–6). The same pattern was determined when diclofenac (1.16% *w*/*w*) was administered (Figure 3, columns 3–4). Ointment antinociceptive activity in phase 2 was significantly lower compared to the reference drug. 

The antinociceptive mechanism of the plant-based ointment was evaluated with the TFT. Data indicate that intraperitoneal (i.p.) administration of naloxone and L-NAME hindered the antinociceptive effect of the ointment in phase 2. On the other hand, glibenclamide showed no effect (Figure 3, columns 7–12). 

Plant-based ointment decreased the NO levels with similar activity as the reference drug diclofenac (1.16%) and restored the NO levels to those found in the basal group in the homogenates from the hind paw of mice in the formalin test (Figure 3b). In addition, plant-based ointment decreased the COX-2 levels with higher activity than diclofenac (1.16%) and almost restored the basal levels of COX-2 (Figure 3d,e). 

### 3.5. Protective Effects of the Ointment on Acute and Chronic TPA-Induced Ear Edema

**Figure 4:**
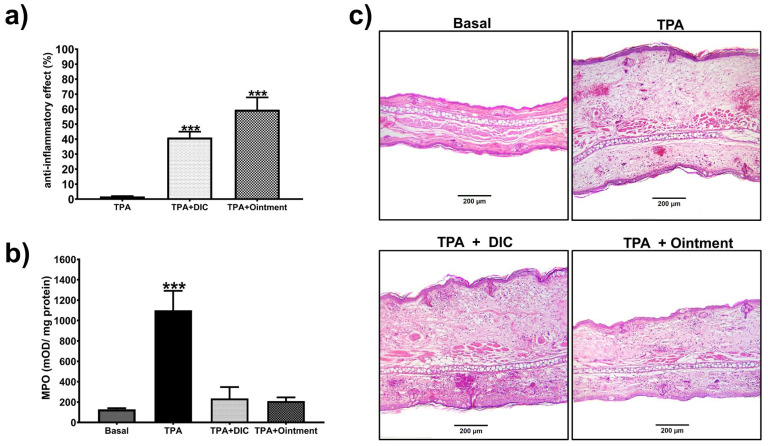
The ointment’s effect on TPA-induced acute ear edema. (**a**) Inhibition effect calculated as a percentage of anti-inflammatory effect of the ointment topically administered to the right ear 60 min before TPA application (2.5 µg in 20 µL acetone). (**b**) The ointment’s effect on MPO activity. (**c**) Representative histological images (H&E staining) of ear biopsies from mice treated with acetone solution (basal), TPA only, treated with the ointment, and diclofenac (DIC) with a dose at 1.16% *w*/*w*. Scale bar is indicated below the histological images. Bars represent mean values (±SEM). n = 8, *** *p* < 0.001 compared to the TPA group in (**a**), *** *p* < 0.001 compared to the basal group in (**b**).

To investigate the anti-inflammatory activity of the ointment, acute and chronic TPA-induced ear edema models were used. In the acute model, topical application of the ointment decreased inflammation by 58%. This value was higher than that determined when the reference drug diclofenac gel (1.16%) was applied. In this case, diclofenac reduced inflammation by 40% (Figure 4a).

The ointment’s anti-inflammatory effect decreased (*p* < 0.05) MPO activity at similar levels to the group treated with diclofenac, obtaining values similar to the basal levels (Figure 4b). The histological pictures in Figure 4c show that topical application of the ointment inhibited the infiltration of inflammatory cells into the skin, significantly reducing ear thickness compared to those where only TPA was administered. In addition, diclofenac gel slightly reduced inflammatory cell infiltration and ear thickness.

**Figure 5:**
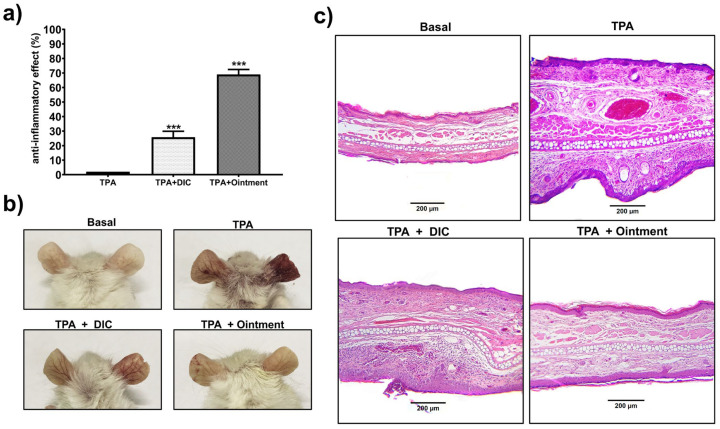
The ointment’s protective effects against chronic TPA-induced ear edema. (**a**) The percentage of anti-inflammatory effect represents ear edema inhibition by topical application of the ointment, which was administered every 48 h for ten days in chronic TPA-induced ear edema (for more information, see the Materials and Methods Section 2). (**b**) Back ear images from mice treated with acetone solution (basal) and treated only with TPA, TPA and diclofenac gel (1.16% *w*/*w*), and TPA with ointment. (**c**) Representative histological images (H&E stain) of ear biopsies from the experimental groups of mice described in (**b**). The scale bar (200 µm) is denoted below each histological image. Bars in (**a**) represent mean values (±SEM). n = 8, *** *p* < 0.001 compared to the vehicle group.

Ointment (basal group) applied in multiple applications (every 48 h for 10 days) in the TPA chronic assay showed no skin irritation in mice. Results of the chronic TPA-induced edema model (Figure 5a) indicate that the plant-based ointment reduced inflammation by 68%; this value was slightly higher than that obtained in the acute edema model, which was 58%. In the chronic TPA-induced edema model, our new product showed an anti-inflammatory effect 2.7 times higher than that obtained with diclofenac gel 1.16% *w*/*w*, which reduced inflammation by 25%. As shown in Figure 5b, the skin protective effects of the ointment were significantly more noticeable. Swelling of and damage to the right ear were significantly reduced by ointment administration (lower right panel) compared to that observed in the ear treated only with the vehicle (upper right panel). Diclofenac gel also visibly reduced the damage induced by TPA (Figure 5b, lower left panel). Histological analysis of the chronic edema model confirmed the anti-inflammatory effect of the ointment, as this product reduced skin monocyte infiltration and tissue thickening more than in tissues treated only with TPA (Figure 5c; upper and lower right panels). Diclofenac 1.16% gel also reduced tissue inflammation in this model (Figure 5c; lower left panel). These data reveal that the ointment exerted a high anti-inflammatory effect in short and long durations of time.

### 3.6. Effect of the Ointment on Inflammatory Mediators

**Figure 6:**
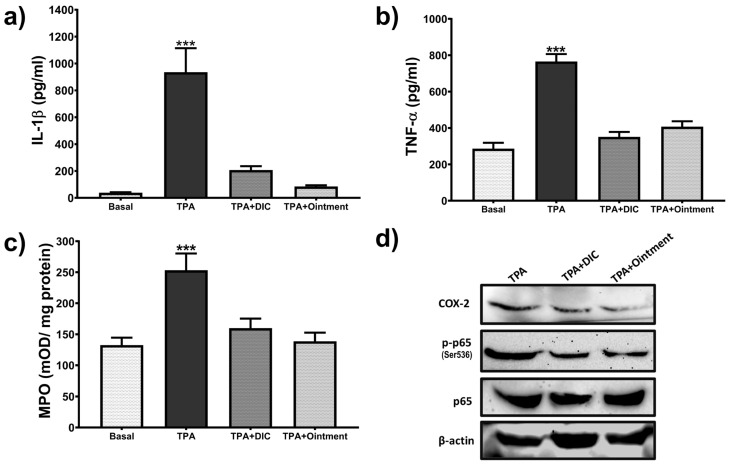
Effect of the plant-based ointment on the production of inflammatory mediators in TPA-induced chronic ear edema. Biopsies from mice treated with acetone solution (basal), TPA, TPA and diclofenac (DIC) 1.16% *w*/*w*, and TPA and the ointment were homogenized in lysis buffer to obtain supernatants used for measurement of IL-1β (**a**), TNFα (**b**), and MPO activity (**c**). (**d**) Representative immunoblots of extracts from homogenized biopsies. *** denotes *p* < 0.05 compared to the basal group.

Topical application of the ointment reduced the levels of pro-inflammatory mediators in the chronic TPA-induced ear edema model. As shown in Figure 6a–c, IL-1β, TNFα levels, and MPO activity significantly increased (*p* < 0.05) in TPA-treated groups compared to the basal group (left ears treated with acetic acid only). Data indicate that ointment application significantly reduced the levels of pro-inflammatory mediators, as their values showed similarity to those observed in the basal groups and those treated with diclofenac gel (1.16% *w*/*w*). The highest anti-inflammatory effect was observed in the groups treated with the plant-based ointment, which reduced the levels of IL-1β. Immunoblot analyses (Figure 6d) also showed that topical application of the ointment slightly decreased NFκB-p65 (Ser536) phosphorylation and COX-2 expression, as the dark bands were comparable to those obtained with diclofenac 1.16% (*w*/*w*).

### 3.7. Docking Study 

The pharmacological results indicate that the coadministration of L-NAME (an iNOS antagonist) or naloxone (an opioid antagonist) reversed the antinociceptive effects of the ointment. Therefore, molecular docking analyzed the possible affinity of ligands (myristic acid, palmitic acid, valeric acid, and stearic acid) with the µ-opioid receptor. The docking results show that the binding energies of all ligands with the μ-opioid receptor (4DKL) were considerably lower than those of the reference drug β-funaltrexamine (−10.56 kcal/mol), which is an irreversible antagonist of the μ-receptor. The lower binding capacity of the ligands with the amino acids could have been due to the null structural relationship that the ligands have with the morphinan drugs that act on the receptor (Table 2). The aliphatic chain of palmitic acid (affinity energy = −5.60 kcal/mol) and myristic acid (affinity energy = −5.61 kcal/mol) interacts with a nonpolar surface, allowing hydrophobic interactions.

**Figure 7:**
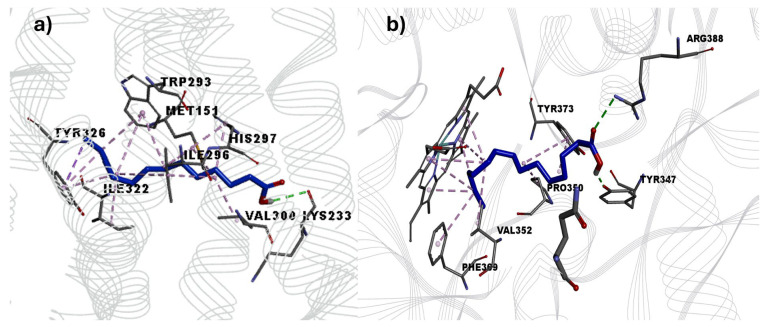
Best poses obtained by molecular docking of myristic acid with the μ-opioid receptor (**a**) and (**b**) iNOS.

Palmitic acid and myristic acid showed nonpolar interactions with Met151, Trp293, Ile296, Val300, and Tyr326 residues. Myristic acid showed a hydrogen bond with the Lys233 residue (Figure 7).

The molecular coupling with iNOS shows that all ligands (myristic acid, palmitic acid, valeric acid, and stearic acid) might have formed hydrophobic interactions with the fatty acid chain oriented towards the center of the catalytic cavity, generating non-polar interactions with the Heme group present in the protein. The affinity energies of palmitic, stearic acid, and myristic acid showed values higher than those of the reference inhibitor ethylisothiourea (ITU) (Table 3). These results suggest that palmitic, stearic, and myristic acids may have an inhibitory effect on iNOS, classifying myristic acid (−6.34 kcal/mol) as the best result since its hydrophobic tail is placed in a parallel arrangement concerning the π cloud of the four pyrrole rings of the porphyrin and with the residues Phe369 and Val352.

**Figure 8:**
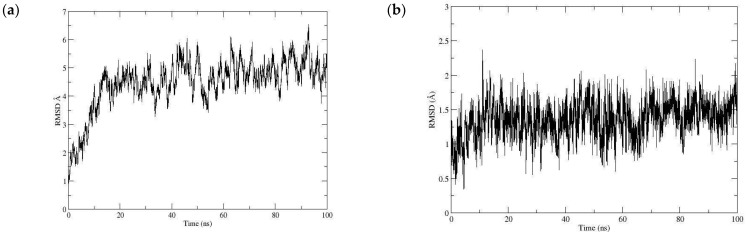
RMSD with the *μ-opioid* receptor of (**a**) the Cα skeleton of the myristic acid with the *μ-opioid* receptor complex (**b**) in the function of the myristic acid ligand.

Once the myristic acid with the μ-opioid receptor interaction was identified, the molecular dynamics technique assessed the stability of the ligand–receptor complex and analyzed the ligand–receptor complex evolution over time. By calculating the root mean square deviation (RMSD), the skeleton of the main chain of the protein showed a deviation of approximately 4.48 Å (Figure 8a), reaching stability after approximately 20 ns. This value indicates that there was a slight fluctuation in the protein. The RMSD value of the ligand aligned with the protein showed an average value of 1.351 Å, indicating the stability of the ligand–receptor interaction (Figure 8b).

**Figure 9:**
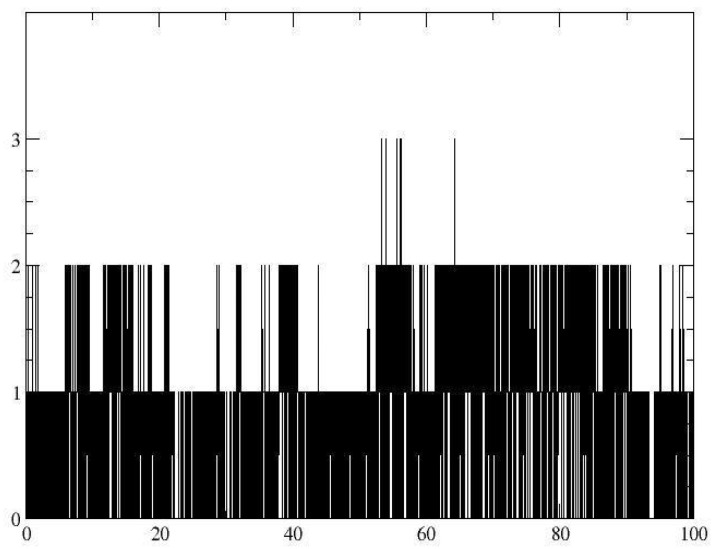
Number of hydrogen bonds formed in 10 ns of stimulation of the myristic acid–μ-opioid receptor complex.

This stability is explained through the analysis of the hydrogen bonds shown in Figure 9, indicating that the hydrogen bond interactions between myristic acid and the *μ-*opioid receptor were Lys233 and Lys303, observed during most of the time of the trajectory and achieving a residence percentage of 22.48% and 53.92%, respectively, of the simulation time. Finally, the free energy binding (ΔG) was estimated by the molecular mechanics Poisson–Boltzmann surface area (MMPBSA) method, obtaining a value of −15.83 kcal/mol and highlighting the highest energetic contributions provided by electrostatic and van der Waals interactions, which is consistent with the nature of myristic acid. These results demonstrate that myristic acid can exert its activity on the μ-opioid receptor.

**Figure 10:**
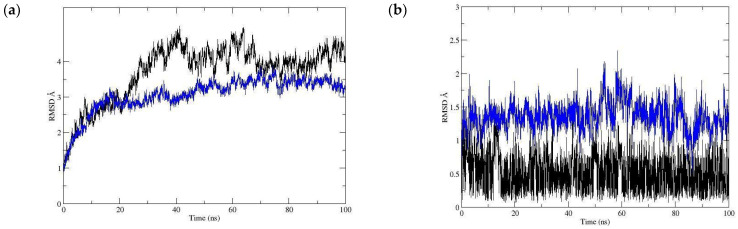
RMSD with iNOS of (**a**) the Cα skeleton of the myristic acid (in blue) with the iNOS complex with ITU (in black) (**b**) in function of myristic acid (blue) and ITU (in black) ligands.

**Figure 11:**
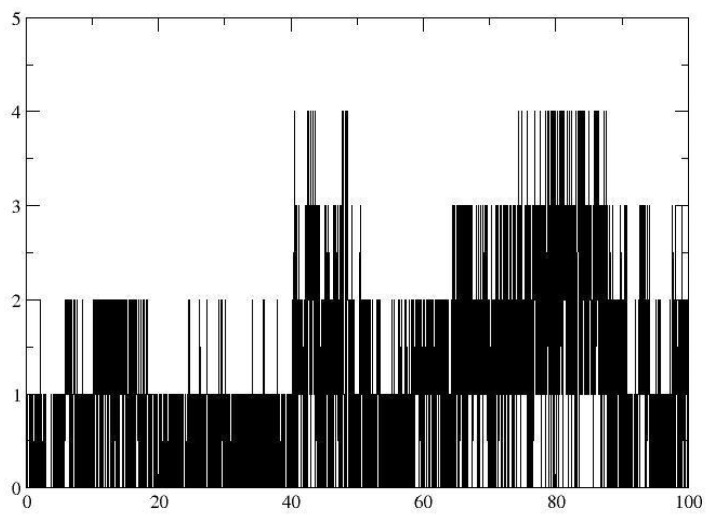
Number of hydrogen bonds formed in 100 ns of stimulation of the myristic acid–4NOS complex.

Given the promising biological and molecular docking results, the analysis of the drug–receptor interaction was continued using the molecular dynamics technique, where the RMSD value was determined, which was initially calculated for the aligned Cα skeleton, obtaining an average value of 3.704 Å for the iNOS-ITU complex and 3.061 Å for the iNOS-myristic acid complex shown in Figure 10a. The fluctuations observed with ITU can be attributed to the high selectivity and conformational changes that this inhibitor can produce in the catalytic site of the protein. When analyzing the stability of the ligand, an RMSD value of 0.503 Å was observed for the iNOS-ITU complex, indicating the high stability and specificity of the selective inhibitor within the catalytic site. The RMSD graph for the complex with MA showed an average RMSD value of 1.355 Å (Figure 10b), indicating the stability of the complex since the trajectory stabilized in the first few seconds of the simulation of the iNOS-MA complex presented hydrogen bond interactions with the residues of Met120, Arg266, and Arg388 with an occupancy during the simulation of 17.38%, 55.10%, and 35.02%, respectively (Figure 11). Finally, the free binding energy ΔGbind = −22.60 kcal/mol for the iNOS–myristic acid complex was more stable than that shown by the reference drug (Δgbind −8.02 kcal/mol). 

## 4. Discussion

This study shows that the plant-based ointment showed antinociceptive and anti-inflammatory properties. Topical application of the ointment resulted in a 50% increase in latency time. This effect was comparable to that obtained when the reference drug ketoprofen gel 2.5% *w*/*w* was used. The antinociceptive effects of the compounds present in the ointment were observed 1–2 h post treatment. The HPT is dependent on rodents’ motor coordination, whereas the TFT is independent of motor coordination [15,16,17]. Both models of nociception are spinally mediated reflexes. In the formalin model, antinociceptive effects of the ointment against chemically induced pain were observed only in phase 2 (15–40 min). This may indicate that the antinociceptive effects are related to the inflammatory phase. The use of naloxone (5 mg/kg), an opioid receptor antagonist, inhibited the antinociceptive effect of the ointment, suggesting that μ receptors located in the nerve endings of the skin could be participating in pain relief [36]. Similarly, L-NAME (20 mg/kg i.p), an antagonist of nitric oxide synthase (NOS), also reversed the antinociceptive effects of the ointment, suggesting that the nitric oxide (NO) pathway and production of pro-inflammatory mediators are involved in the inhibition of pain induced by the ointment [37,38]. On the contrary, glibenclamide, an antagonist of ATP-sensitive K^+^ channels, did not show a reversible effect on the ointment antinociceptive activity. Plant-based ointment decreased the levels of COX-2 and NO in the homogenates from the hind paw of mice in the formalin test. These results confirm that the NO pathway and COX-2 are involved in the antinociceptive actions of plant-based ointment. 

Ointment anti-inflammatory effects were assessed using acute and chronic TPA-induced ear edema models. Ointment application decreased skin inflammation induced by TPA after 6 h. This effect resulted in a reduction in ear swelling, monocyte infiltration, and MPO activity. The observed anti-inflammatory effect of the ointment was higher (70%) in the chronic TPA-induced ear edema model than that determined in the acute inflammation model (55%). Ten days after treatment, the ointment protected the ear skin from scab formation. Ear skin in mice treated only with TPA showed severe tissue damage. Ear swelling reduction resulted in a decrease in immune cell infiltration, and this effect was higher in the ointment-treated group than that determined in mice administered with diclofenac 1.16% gel. The anti-inflammatory activity of the ointment was reflected as a decrease in pro-inflammatory mediators such as IL-1β and TNFα and the activity of MPO, reaching almost basal levels. Anti-inflammatory activity likely resulted from the suppression of the classic NF-kB (p65) inflammation pathway. COX-2 is an enzyme responsible for producing prostaglandins and is involved in pain and fever induced by pro-inflammatory mediators from the NF-κB pathway [37,39]. This fact explains the inhibition of this pathway and the consequent attenuation in the expression of COX-2 induced by the ointment.

Previous research with in vivo and in vitro models demonstrated that plants used in the ointment studied in the present investigation displayed antinociceptive and anti-inflammatory effects. For instance, the intraperitoneal administration of EG essential oil (45 mg/kg) produced antinociceptive effects in the FT and the acetic acid-induced abdominal writhing test, with the potential participation of the opioidergic system [4]. In lipopolysaccharide (LPS)-stimulated murine macrophages, EG essential oil (0.02%) showed anti-inflammatory activity. Also, EG essential oil downregulated NOD-like receptor protein (NLRP) 3 inflammasome and reduced IL-1β production, hindered NF-κB phosphorylation, and inhibited pattern recognition receptor pathways [3,4]. Curcumin polyphenols are one of the main components of CL, and the pharmacological effects of curcumin have also been extensively studied. Oral curcumin showed antinociceptive properties in the HPT and TFT tests, inhibiting MPO activity, recruitment of leukocytes, production of cytokines via NF-κB, and suppressing neuroinflammation caused by brain injury in animals [38,40,41,42,43]. These results are comparable to those obtained in the present study with our new plant-based ointment. Glycolic extracts from HV bark produced anti-inflammatory effects in a skin inflammation model where human keratinocytes (HaCAT) infected by *Cutibacterium acnes* were used. It was observed that NFκB and IL-6 levels decreased (IC_50_ = 136.90 ± 17.22 μg/mL) [7]. Also, EP exhibited nephroprotective activity by preventing oxidative damage, suppressing the expression of NLRP3 and toll-like receptor (TLR) 4, and inhibiting cytokines such as TNFα and IL-6 as well as the production of reactive oxygen species (ROS) [8,9,44]. Ginger, another ointment component, presents cardioprotective effects against inflammation and fibrosis, and as an analgesic enhancer evaluated in the HPT test [11,12]. Ginger extracts in hydrogel applied to rat skin reduced the production of ROS and prostaglandins E2 (PGE2), and treatments through liposomes combined with metformin inhibited IL-22 and TNFα and improved psoriatic skin lesions [11,12]. These results validate the data, which indicate that this ointment protects the skin against tissue damage.

FAs identified in the plant extracts of the ointment can reduce inflammation and pain. In experiments with rodents, the injection of FAs (ARA 3000 beta) showed promising results for the treatment of osteoarthritis and arthritis by modulating inflammation and mediators such as PGE2, NO, and metalloproteases [45,46]. In vitro and in vivo studies also demonstrated that palmitic acid combined with oleic acid reduced inflammatory markers such as COX-2, TNFα, IL-6, and IL-12 in LPS-stimulated macrophages, and reduced inflammatory parameters in models of dextran sulfate sodium (DSS)-induced colitis in mice [47]. In addition, the mixture of palmitic acid (PA) and myristic acid (MA) enhanced anti-inflammatory effects in skin infected with *Candida* spp. in zebrafish models [48]. Oral administration of stearic acid (SA) displayed protective effects in cholestasis-induced liver injury in rats, decreasing the accumulation of leukocytes, MPO activity, activation of NF-κB, and production of transforming growth factor-beta 1 (TGF-β1) [49]. SA is one of the main components of *Cardiospermum halicacabum*, a plant used in traditional Indian medicine to treat conditions such as abdominal pain, skin disease, cough, rheumatism, and hyperthermia [50]. SA and PA are FAs that are safe ingredients with moisturizing properties used in cosmetics and skincare products [51]. Supplements containing FAs in coconut oil reduced inflammation and attenuated chemotherapy toxicity in rats [52]. These data may explain the pharmacological activities of the FAs present in our new ointment. 

Aliphatic hydrocarbons (docosene, heneicosane, and others) were also identified in the new ointment. Precedent research has determined that aliphatic hydrocarbons are one of the main components of *Crepis foetida*, a plant with in vitro anti-inflammatory activity [53]. Aliphatic hydrocarbons are also present in essential oils of *Amomum subulatum* fruits. These compounds displayed anti-inflammatory properties in xylene-induced edema models [54]. 

Palmitic acid and myristic acid showed nonpolar interactions with Met151, Trp293, Ile296, Val300, and Tyr326 residues. Those interactions were previously reported with β-funaltrexamine [55]. Myristic acid showed a hydrogen bond with the Lys233 residue, a key residue within the receptor recognition site in the *μ-*opioid receptor [56]. Furthermore, the Lys233 H-bond interaction has been reported with other antagonists, such as naltrexone [57]. Therefore, this polar interaction could be essential for maintaining a stable ligand–receptor interaction to generate a possible nociceptive effect with other recognition pathways. 

The conformation of myrisitc acid can interact with the amino acids of the first layer of the catalytic site of iNOS through hydrogen bonds with the residues Arg388 and Tyr347, key residues in the activity of the human iNOS enzyme. These interactions cause fluctuations in the neighboring residues that can produce an open Gln conformation in iNOS [58]. Previous reports showed a close relationship between myristic acid and the enzyme endothelial nitric oxide synthase (eNOS), where myristic acid is incorporated into the *N*-terminal residue through N-myrostylication [59]. The regulation of this enzyme is due to the absorption of myristic acid through the class B scavenger protein CD36, which modulates the signaling for the NO expression through eNOS [60]. Recent studies using in vivo models reported that myristic acid significantly decreased iNOS levels in rat testes [61]. 

Our previous work showed that myrisitic acid exerted antinociceptive activity (ED_50_ = 32 mg/kg p.o.) in the formalin test in mice, with the possible participation of the nitrergic system [14]. In the previous study, we hypothesized that myristic acid might compete with *L*-NAME for the binding site of iNOS. The docking study corroborated the binding affinity of myristic acid and iNOS. These results of the free binding energy ΔGbind = −22.60 kcal/mol for the iNOS-myristic acid complex are consistent with those obtained from biological tests and the previously proposed mechanism of action, so the antagonistic inhibition of myristic acid at the catalytic site of induced nitric oxide synthase can be a route of action.

Some of the study’s limitations included the lack of in vitro dissolution and permeation studies and long-term studies to evaluate skin irritation tests. The combination of plant extracts in the new ointment showed anti-inflammatory and antinociceptive activities comparable to those of the two reference drugs, diclofenac and ketoprofen. As previously explained, each plant contains metabolites that act through different mechanisms (i.e., the opioid system and inhibiting pro-inflammatory mediators). When plants with distinct mechanisms of action are combined, the pharmacological effects can be improved, as with this ointment. Herein, we propose to perform clinical studies to evaluate the analgesic and anti-inflammatory effects of the new ointment in patients.

## 5. Conclusions

An ointment containing extracts of *E. globulus*, *C. longa*, *H. virginiana*, *E. purpurea*, and *Z. officinale* displayed antinociceptive and anti-inflammatory properties. According to the results of in vivo heat-induced and chemical-induced noxious stimulus experiments, the ointment’s antinociceptive effects were comparable to those induced by ketoprofen gel 2.5% *w*/*w*. It is likely that antinociceptive activities are potentially mediated by the decrease in COX-2 and NO levels, and possible participation of the opioidergic system. 

The ointment also showed anti-inflammatory activity in the acute and chronic TPA-induced ear edema models. These effects were characterized by a reduction in ear swelling and monocyte infiltration in the acute model of inflammation, whereas in the chronic model, the mechanism of action relied on a decrease in IL-1β and TNFα levels, a reduction in MPO activity, and a decrease in NF-κB (p65) and COX-2 expression. The in-silico study showed that myristic acid, one of the compounds identified in the ointment’s plant mixture, is an antagonist at the catalytic site of induced nitric oxide synthase. This finding corroborated the pharmacological experiments carried out in the formalin test with the coadministration of L-NAME.

Further preclinical studies using long-term assays in animal models are necessary to evaluate the pharmacological (anti-inflammatory and antinociceptive) and toxicological (skin irritation test) effects of this plant-based ointment. Clinical studies can be considered after these assessments are carried out.

## 6. Patents

A patent pending method (MX/a/2024/008251) to obtain the plant extracts is reported.

## Figures and Tables

**Table 1:** Compounds identified in the ointment using GC-MS.

No.	Compound	* RT	TRI	ERI	%RE
1	1-Ethoxy-1-(trimethylsiloxy)octane	7.59	1311	1269	3.20
2	5-(Cyclohexylmethyl)-2-pyrrolidinone	8.64	1484	1485	0.07
3	3,4-Dimethylbenzoic acid, trimethylsilyl ester	8.68	1493	1489	0.22
4	Trimethyl-[2-[2-(2-trimethylsilyloxyethoxy)ethoxy]ethoxy]silane	8.72	1489	1493	0.27
5	α-[3′-(Trifluoromethyl)benzyl]-γ-butyrolactone	8.81	1507	1501	0.40
6	Ethisolide	8.94	1540	1514	1.69
7	2-Hexyldecanol	9.21	1504	1540	2.39
8	Ethyl phthalate	9.72	1578	1588	0.63
9	2-Methyl-6-(4-methylphenyl)-2-hepten-4-one	10.48	1660	1667	0.42
10	2-Methylhexadecane	10.69	1664	1689	1.50
11	Myristic acid, trimethylsilyl ester	12.07	1842	1840	0.11
12	n-Nonadecane	12.63	1900	1906	0.32
13	D-Mannitol, 1,2,3,4,5,6-hexakis-O-(trimethylsilyl)-	12.71	1979	1916	3.18
14	n-Pentanoic acid, trimethylsilyl ester	12.90	1942	1939	0.15
15	Sulfurous acid, butyl nonyl ester	12.99	1937	1948	0.57
16	1-Trimethylsiloxyhexadecane	10.01	1965	1951	0.71
17	Palmitic acid, trimethylsilyl ester	13.71	2039	2022	0.83
18	2-Hydroxy-2-methyl-N-phenyl-1,4-dioxane-3-carboxamide	14.32	2043	2071	1.37
19	Trimethylsilyl heptadecanoate	14.40	2087	2083	0.19
20	n-Heneicosane	14.64	2100	2096	0.19
21	Docos-1-ene	14.94	2192	2187	0.23
22	cis-9-Octadecenoic acid, trimethylsilyl ester	15.03	2208	2217	0.41
23	Stearic acid, trimethylsilyl ester	15.20	2236	2284	2.15
24	2,6,10,15,19,23-Hexamethyl-2,6,10,14,18,22-tetracosahexaene	19.02	3548	3587	1.10
25	Methyl 9-desoxomesopyrochlorophllide	19.32	3698	3690	0.22
26	3β-ACETOXY-CHOLEST-6-ENO-[7,6-D]-2′-PHENYLOXAZOL	21.89	4598	4573	0.54

* RT—retention time; TRI—theoretical retention index; ERI—experimental retention index; %RE—relative error.

**Table 2:** Energy interaction shown by the ligands with μ-opioid receptor (4DKL) and possible interactions.

Protein	Ligand	Affinity Energy (kcal/mol)	Type of Interactions
4DKL Morphine (reference drug) −10.56 kcal/mol	Stearic acid	−6.13	π-sigma Trp293, His297, Tyr226 Alkyl/pi-alkyl: Met151, Ile296, Ile322, Val300
	Palmitic acid	−5.60	H-bond: TYR148 π-Sigma: Trp293 Alkyl/pi-alkyl: Met151, Val236, Ile296, His297, Val300
	Valeric acid	−3.26	H-bond: TYR148 Alkyl/pi-alkyl: Val236, His297, Val300
	Myristic acid	−5.61	H-bond: **Lys233** π-Sigma: Tyr326 Alkyl/pi-alkyl: Met151, Trp293, Ile296, His297, Val300, Ile322

**Table 3:** Energy interaction shown by the ligands with the *i*NOS receptor (4NOS) and other molecular interactions.

Protein	Ligand	Binding Energy (kcal/mol)	Interactions
4NOS Ethilisothiourea (referencia) −4.75 kcal/mol	Stearic acid	−6.69	H-bond: Tyr347 Alkyl/π-Alkil: Pro350, Val352, Phe369, Tyr372, HEM510
	Palmitic acid	−6.60	H-bond: Tyr347, Ala351 Alkyl/π-Alkil: Pro350, Val352, HEM510
	Valeric acid	−3.92	H-bond: Phe369 Alkyl/π-Alkil: Pro350, HEM510
	Myristic acid	−6.34	H-bond: Tyr347, Arg488 Alkyl/π-Alkil: Pro350, Val352, Phe369, Tyr373, HEM510

## Data Availability

The original contributions presented in the study are included in the article, further inquiries can be directed to the corresponding author.
